# Retinopathy caused by a primary immune regulatory disorder - the spectrum of *AIRE*-associated retinopathy: case series and literature review

**DOI:** 10.1038/s41433-026-04365-9

**Published:** 2026-04-09

**Authors:** Mohammad Anas, Andrew C. Browning, Siying Lin, Omar A. Mahroo, Andrew R. Webster, Margaret Reynolds, Tatyana Milman, Ralph C. Eagle, Richard Vile, Marc Toso, Gregory S. Hageman, Hossein Nazari, Erik J. Van Kuijk, Jose S. Pulido

**Affiliations:** 1 Department of Ophthalmology, Wills Eye Hospital, Thomas Jefferson University, Philadelphia, PA USA; 2 Department of Ophthalmology, Guy’s & St Thomas’ NHS Foundation Trust, London, UK; 3 UCL Institute of Ophthalmology, University College London, London, UK; 4 Newcastle Eye Centre, Royal Victoria Infirmary, Newcastle upon Tyne, UK; 5 NIHR Biomedical Research Centre at Moorfields Eye Hospital and the UCL Institute of Ophthalmology, London, UK; 6 Division of Evolution, Infection and Genomics, School of Biological Sciences, Faculty of Biology, Medicine and Health, University of Manchester, Manchester, UK; 7 Manchester Centre for Genomic Medicine, Saint Mary’s Hospital Manchester University NHS Foundation Trust, Manchester, UK; 8 Department of Ophthalmology and Visual Sciences, Washington University in St. Louis, St. Louis, MO USA; 9 Department of Pathology, Wills Eye Hospital, Sidney Kimmel College of Medicine at Thomas Jefferson University, Philadelphia, USA; 10 Departments of Molecular Medicine and Immunology, Mayo Clinic, Rochester, MN USA; 11 Joan Reece Chair of Immuno-oncology, Comprehensive Cancer Centre, School of Cancer and Pharmaceutical Sciences, and School of Immunology and Microbial Sciences, Kings College London, London, UK; 12 John A. Moran Eye Centre, Department of Ophthalmology & Visual Sciences, University of Utah, Salt Lake City, UT USA; 13 Department of Ophthalmology and Visual Neurosciences, University of Minnesota, Minneapolis, MN USA

**Keywords:** Disease genetics, Mutation, Endocrine system and metabolic diseases

## Abstract

**Background/Objective:**

Retinal involvement in autoimmune polyendocrine syndrome type 1 (APS1), a rare monogenic autoimmune disorder caused by mutations in the *AIRE* gene, is increasingly recognised but remains poorly defined. Prior reports suggest a variable phenotype, ranging from mild changes to severe vision loss, often presumed untreatable. We explored the range of retinal phenotypes associated with *AIRE* gene deficiency in a multicentre case series of patients with APS1.

**Methods:**

We performed a retrospective case note review of patients with molecularly confirmed APS1 from tertiary ophthalmic centres. Clinical history, multimodal retinal imaging, electrophysiology, genetic data, and treatment regimens were analysed. Histopathology was available in one case postmortem.

**Results:**

Records were reviewed from five unrelated female patients. Median age was 14 years at onset of ocular involvement and 33 years at most recent follow up. Some findings from two cases have been previously reported. Three distinct pathogenic *AIRE* variants contributing to biallelic genotypes were observed. Retinal findings ranged from structurally and functionally normal to advanced degeneration. One patient demonstrated sharp zonal atrophy on histopathology. Inflammatory features predominated in two cases, both showing durable vision preservation with periocular or systemic immunomodulation. One patient demonstrated four years of disease stabilisation with rituximab. No consistent genotype-phenotype correlation emerged.

**Conclusion:**

*AIRE*-associated retinopathy encompasses a diverse spectrum, from clinically silent to profound degeneration. Early, targeted immunomodulation might preserve vision in selected cases. These findings advocate for ophthalmic surveillance in APS1, and support further investigation into predictive biomarkers and possible tailored immunotherapy in this vision-threatening autoimmune disorder.

## Introduction

Autoimmune polyglandular syndrome type 1 (APS1; OMIM #240300), also referred to as autoimmune polyendocrinopathy-candidiasis-ectodermal dystrophy (APECED), is a rare autosomal recessive disorder resulting from biallelic pathogenic variants in the autoimmune regulator (*AIRE*) gene [1]. The *AIRE* gene encodes a 545-amino acid proline-rich protein that is essential for the establishment of central immune tolerance [2].

*AIRE* is predominantly expressed in medullary thymic epithelial cells (mTECs) within the thymus, where it governs the ectopic transcription of a broad array of tissue-restricted antigens. This ectopic expression is critical for the negative selection of autoreactive thymocytes. Loss of AIRE function impairs this negative selection checkpoint, facilitating the survival of high-affinity self-reactive T cells and, secondarily, B cells, which in turn predisposes to systemic autoimmunity and is one of the primary immune regulatory disorders [3].

Clinically, APS1 is classically defined by the triad of chronic mucocutaneous candidiasis, hypoparathyroidism, and primary adrenal insufficiency [4]. However, the phenotypic spectrum is considerably broader, encompassing a range of autoimmune endocrinopathies, including autoimmune thyroid disease, type 1 diabetes mellitus, gonadal failure, and autoimmune hepatitis, as well as non-endocrine manifestations such as vitiligo and enamel hypoplasia.

Ocular involvement has increasingly been recognised as part of the APS1 phenotype, with autoimmune retinopathy emerging as a significant yet under-characterised manifestation. Circulating antiretinal autoantibodies have been implicated in disease pathogenesis, although their exact role remains incompletely defined [5]. Reports in the literature indicate a variable prevalence and heterogeneity in retinal findings among individuals with pathogenic *AIRE* gene variants [6].

In this multicentre case series, we present a cohort of individuals with genetically confirmed AIRE deficiency, exhibiting a range of retinal involvement from normal findings to profound degenerative changes. This study aims to describe in detail the phenotype in cases of *AIRE*-associated retinopathy and integrate these observations with a review of the existing literature.

## Methods

### Case identification

Cases of *AIRE*-associated disease were collected from selected ophthalmic centres. In the United States, these were at Washington University at St Louis, the John Moran Eye Center, Salt Lake City and the University of Minnesota. In the United Kingdom, these were Moorfields Eye Hospital in London and the Eye Department of the Royal Victoria Infirmary, Newcastle upon Tyne (UK); the latter institution is a referral centre for APS1. Data reviewed included clinical history, multimodal retinal imaging (which included, where available, colour or pseudocolour fundus images, optical coherence tomography (OCT), fundus autofluorescence (FAF), fluorescein angiography), electrophysiology, genotype, treatment regimens and visual outcomes. Histopathology was available in one case postmortem.

### Literature search

Previously reported cases of retinal involvement in association with APS1 were identified from the literature (using search terms including Autoimmune polyglandular syndrome type 1, APS1, AIRE, retina). Key features extracted from prior reports, where available, included patient demographics, AIRE variants, retinal and systemic findings, treatments and ocular outcomes.

## Results

### Case series

We report five unrelated individuals (five females) from separate tertiary centres, each with molecularly confirmed diagnoses of APS1. The median age at first ocular symptom or sign was 14 years (range 3–20 years) and at most recent follow-up was 33 years (range, 19–69 years), with a median ophthalmic follow-up duration of 14 years (range, 5–57 years). Case 2 and 5 have previously reported data [7–10]. Three distinct *AIRE* variants were identified across the cohort: the recurrent frameshift variant (NM_000383.4) c.967_979del; p.(Leu323Serfs*51) the founder nonsense variant c.769C>T; p.(Arg257*), and a previously reported deletion c.1265del; p.(Pro422Leufs*58) [11, 12]. Retinal involvement was observed in four patients. Notably, one patient (Case 1) exhibited no anatomical or functional evidence of retinopathy over five years of high-resolution imaging and electrophysiological surveillance.

Key systemic and ocular features for all five patients are summarised in Table 1.

**Table 1:** Systemic profile, management and follow up of the five patients in our case series.

ID	Age (years)/ sex	*AIRE* genotype	Retinal Phenotype	First ocular event (age)	Visual acuity at most recent follow up	Systemic APS1 manifestations	Immunotherapy given	Follow-up (years)
Case 1	19 F	Homozygous c.967_979del; p.(Leu323Serfs*51)	No outer retinal involvement; mild papilledema	14 y - papilledema	20/20 both eyes	Classic triad (Chronic mucocutaneous candidiasis, hypoparathyroidism, adrenal insufficiency)	Systemic replacement steroids (hydrocortisone 25 mg daily split into 3 doses; fludrocortisone 300 mcg daily)	5
Case 2^a^	25 F	Homozygous c.769C>T; p.(Arg257*)	Right disc swelling. Zonal areas of outer retinal degeneration (radiating from optic disc in RE).	~20 y – blurred vision	20/40 RE, 20/40 LE	Classic triad	Systemic replacement steroids (hydrocortisone 15 mg daily split into 2 doses); fludrocortisone 100 mcg daily).	2
Case 3	60 F	Compound heterozygous c.967_979del; p.(Leu323Serfs*51)/ c.1265del; p.(Pro422Leufs*58)	Areas of peripheral chorioretinal degeneration (multifocal in the right eye; more confluent in the left eye); vitreous debris; left epiretinal membrane.	3 y - nyctalopia	20/30 RE, 20/40 LE	Triad plus renal stones, type 1 diabetes	,5 right eye sub-Tenon’s triamcinolone injections between the ages of 46 and 53; replacement steroids (hydrocortisone 20 mg daily and fludrocortisone 200 mcg daily)	57
Case 4	69 F	Homozygous c.967_979del; p.(Leu323Serfs*51)	Widespread outer retinal degeneration involving central and peripheral retina with marked perivascular degeneration in the far periphery (both eyes).	12 y - nyctalopia	Light-perception BE	Adrenal insufficiency, hypoparathyroidism, ovarian failure	Replacement steroids (further details not available)	56
Case 5^a^	33 F	Compound heterozygous c.967_979del; p.(Leu323Serfs*51)/ c.769C>T; p.(Arg257*)	Widespread outer retinal degeneration resembling retinitis pigmentosa (with sparing of the central macula, within a hyperautofluorescent ring) in both eyes.	19 y - field constriction	20/25 RE, 20/30 LE	Triad, ectodermal changes	Cyclosporine 100 mg daily; mycophenolate mofetil 2500 mg daily; prednisone 7.5 mg daily; beta-carotene 25,000 International units; lutein 20 mg; infliximab infusion every 8 weeks. Previously given Rituximab over 4-year period.	14
Median	33 5/5 F			14 y	20/20			14

Treatments refer to current/most recent regimes unless otherwise stated. Full treatment details for Case 4 are not available.

*RE* right eye, *LE* left eye, *BE* both eyes.

^a^These cases have prior published data [7, 8].

#### Case 1

This patient, a 19-year-old woman, fulfilled the diagnostic triad of APS1 in childhood. Genetic testing confirmed a biallelic homozygous pathogenic *AIRE* frameshift deletion variant: c.967_979del; p.(Leu323Serfs*51), inherited in *trans* from both parents, consistent with autosomal recessive autoimmune polyendocrinopathy with candidiasis and ectodermal dysplasia. At 14 years old, she was diagnosed with idiopathic intracranial hypertension (IIH). Fundoscopy revealed bilateral optic disc hyperaemia, but foveal reflexes were preserved, and best-corrected visual acuity (BCVA) was 20/20 in both eyes. Other than disc swelling, ocular examination and multimodal retinal imaging remained normal (Supplementary Fig. 1). Peripapillary retinal nerve fibre layer (RNFL) OCT revealed increased thickness (mean: 164 µm OD, 141 µm OS).

Her IIH was medically managed with acetazolamide under neurology supervision. Serial OCT-RNFL scans documented progressive normalisation of RNFL thickness, followed by mild thinning to the low-normal range (final global RNFL: 103 µm OD, 99 µm OS). No visual field defects were detected. Throughout five years of surveillance, BCVA has remained 20/20 bilaterally. She reports no nyctalopia, photopsia, or colour vision disturbances. Full-field electroretinogram (ERG) at the age of 18 years was entirely within reference limits.

#### Case 2

This was a 25-year-old woman with biallelic disease-causing *AIRE* variants, specifically the founder nonsense variant c.769C>T; p.(Arg257*). She had been diagnosed clinically at 11 years old with the classical APS1 triad. The abnormal areas of histology were previously reported [8]. Long-term corticosteroid replacement with hydrocortisone and fludrocortisone was initiated at diagnosis; no additional immunosuppressive therapy was employed.

At 23 years old, the patient reported blurred vision for some years, and ocular examination revealed corneal epithelial erosions and cataracts. Visual acuities were 20/125 (right) and 20/50 (left). Fundus examination revealed right optic disc swelling (MRI showed signs of raised intracranial pressure), subtle peripapillary retinal pigment epithelium (RPE) mottling and peripheral pigment clumping (fundus photographs from the age of 23 years are in the previous report [8]), with preserved macular appearance. She was treated with azetazolamide and underwent right eye cataract surgery and reported improvement in vision. Her most recent recorded visual acuities were 20/40 in both eyes. The patient was subsequently lost to follow up and died at 25 years of age from cardiac arrest due to electrolyte abnormalities.

**Fig. 1:**
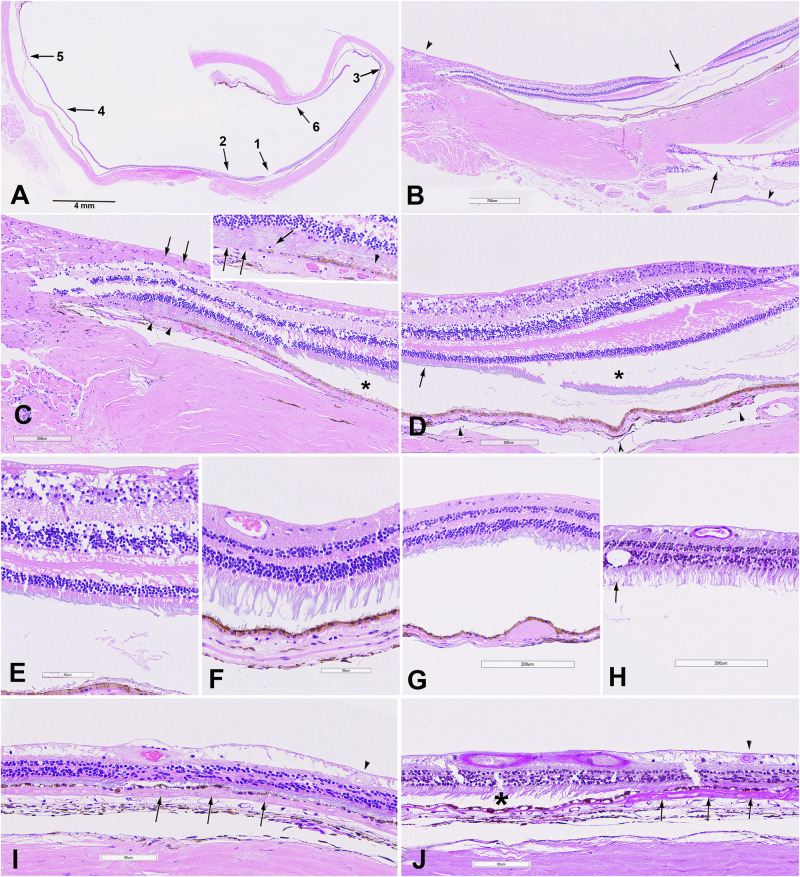
Case 2, histopathologic findings of the left retina, RPE, and choroid outside of pseudo-retinitis pigmentosa changes reported previously [18]. **A** Overview of posterior segment at scanning magnification with regions of interest magnified in panels (**B**–**J**). **B** Higher magnification of region designated as “1” and “2” in (**A**) through fovea (arrow) and optic nerve (arrowhead). Inset: Higher magnification of fovea with likely artefactual pseudo-cystoid changes in the retina (arrow) and artefactual detachment of photoreceptors (arrowhead). **C** Higher magnification of optic nerve and adjacent temporal retina shows mild retinal nerve fibre layer (RNFL) thinning associated with mild gliosis (arrows); RPE is focally atrophic and hypopigmented (arrowheads) and overlying retina shows outer retinal folds/tubulations. The outer retina is otherwise well-preserved and is focally artifactually detached (asterisk). The choroid shows fewer melanocytes, compared to choroid in other locations. The RPE and choroid changes correspond to the atrophic pigmentary changes described clinically [8]. The inset highlights region of RPE atrophy and hypopigmentation (2 arrows), retinal tubulations (single arrow), and subretinal proteinaceous debris (arrowhead), compatible with history of optic disc oedema [8]. **D** Higher magnification of the nasal fovea shows largely unremarkable retina with mild RNFL thinning, well-preserved outer retina including photoreceptor segments (arrow), artefactual retinal detachment, and artefactual detachment of photoreceptor segments (asterisk). Note more pronounced pigmentation in the choroid (arrowheads). **E** Higher magnification of region designated with arrow in (**D**). **F** Post-equatorial temporal retina, corresponding to region “3” in (**A**) is largely unremarkable with preserved photoreceptor segments and other layers. **G** Nasal equatorial posterior segment, corresponding to region “4” in (**A**) the figure (**A**) shows a soft drusenoid sub-RPE deposit. Subtle degenerative changes in outer photoreceptor segments in overlying artefactually detached retina may also be an artefact. **H** Peripheral nasal retina corresponding to region “5” in (**A**) close to ora serrata, shows the posterior edge of typical peripheral cystoid degeneration (arrow) and is otherwise unremarkable with good preservation of photoreceptor segments and artefactual detachment. **I** Peripheral temporal retina, corresponding to region “6” in (**A**) shows focal chorioretinal adhesion with patchy outer retinal and RPE atrophy, choriocapillaris atrophy, and neovascularisation within diffuse soft drusen (arrows). Sclerosed vessel is present in the inner retina (arrowhead) and RNFL shows degenerative changes. **J** Corresponding Periodic acid-Schiff-stained preparation shows neovascular membrane within diffuse drusen (arrows) with overlying patchy RPE and outer retinal atrophy, sclerosed retinal vessel (arrowhead) and underlying choriocapillaris atrophy. The more posterior unremarkable retina is artefactually detached (asterisk). [haematoxylin-eosin (**A**–**G**, **I**) PAS (**H**, **J**)].

As previously reported, gross examination of both globes revealed a pattern of zones of outer retinal atrophy, some of which were contiguous with the optic disc, sparing the fovea and histological analysis of the affected areas demonstrated complete loss of photoreceptors, RPE, and choriocapillaris, with RPE hyperplasia, and reactive gliosis reminiscent of retinitis pigmentosa [8]. We now report and demonstrate that the adjacent extra-lesional retina was structurally preserved in the left eye (Fig. 1, stained with haematoxylin and eosin). Importantly, there was no evidence of active intraocular inflammation - no lymphocytic infiltration or granuloma formation - suggesting the absence of high-grade uveitis at the time of death. Furthermore, there was a type 1 choroidal neovascular membrane in the peripheral temporal retina in close proximity to the area with photoreceptor loss (Fig. 1J).

This case illustrates a manifestation of *AIRE*-associated retinopathy characterised by localised, progressive zonal degeneration with preserved left eye central vision until death, yet histologically severe photoreceptor and RPE loss in affected areas.

#### Case 3

This case involves a 60-year-old woman with early-onset nyctalopia, first noted at 3 years old, followed by bilateral cataract needling at the age of 4 years due to hypocalcaemia-induced cataracts, resulting in aphakia. By 6 years of age, she had developed multiple systemic features consistent with APS1, including congenital hypoparathyroidism, Addison disease (managed with hydrocortisone and fludrocortisone), type 1 diabetes mellitus, nephrolithiasis-associated renal impairment, and chronic mucocutaneous candidiasis. She was not receiving systemic immunosuppression.

Molecular genetic testing revealed two pathogenic *AIRE* frameshifting deletions: c.967_979del; p.(Leu323Serfs*51) and c.1265del; p.(Pro422Leufs*58). Although phasing could not be established, the clinical phenotype was fully consistent with autosomal recessive APS1.

The patient remained ophthalmologically stable until 25 years old, when anterior and intermediate uveitis were diagnosed, treated with periocular corticosteroid therapy. By the age of 31 years, she had been diagnosed with “retinitis pigmentosa” at an external clinic; however, her phenotype evolved to asymmetric multifocal chorioretinal scarring with peripheral pigment clumping. Visual acuity with contact lenses was 20/30 bilaterally by 40-years-old.

**Fig. 2:**
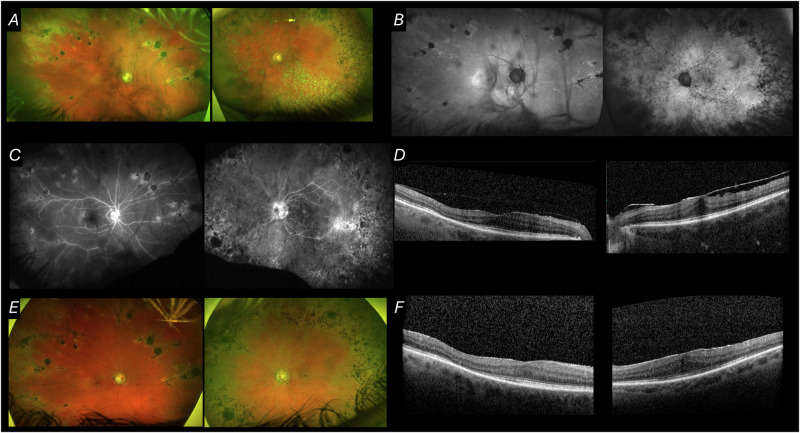
Multimodal imaging from Case 3. Ultra-widefield pseudocolour (**A**) autofluorescence (**B**) fluorescein angiography (**C**) and OCT images (**D**) from right and left eyes aged 52. The fluorescein angiograms were at 31 s and 2 min 10 s for right and left eyes, respectively. Follow-up ultra-widefield pseudocolour (**E**) and OCT (**F**) images aged 60.

Retinal imaging, at 52 and 60 years of age, is shown in Fig. 2A–D, aged 52; E, F, aged 60. At 52 years old, multifocal areas of chorioretinal scarring were visible clinically and on FAF (asymmetric and more confluent in the left eye), with fluorescein angiography showing areas of nonperfusion and disc leakage. Macular OCT showed temporal parafoveal outer retinal atrophy in the right eye and an epiretinal membrane in the left. She subsequently developed bilateral granulomatous uveitis. Ocular hypertension was diagnosed at 54 years old.

Surgical interventions included right pars plana vitrectomy at 55 years old for vitreous debris and left vitrectomy with epiretinal membrane peel at 59 years old. At most recent follow up (60 years old), she maintains visual acuity of 20/30 (OD) and 20/40 (OS) with contact lens correction (imaging shown in lower panels of Fig. 2).

Systemic immunosuppression was not used for this individual, management included topical lubricants (Hylo-Forte) and intraocular pressure control with topical bimatoprost/timolol eye drops. Vitritis flares were treated with sub-Tenon’s triamcinolone (5 injections between 46 and 53 years of age). Intraoperative PCR testing for viral pathogens during vitrectomy was negative. Anti-retinal antibody testing was negative (recoverin, carbonic anhydrase II and alpha enolase).

#### Case 4

This case is a 69-year-old woman who presented with nyctalopia at 12 years old. She was subsequently followed under the diagnosis of “retinitis pigmentosa/retinal dystrophy”. Systemic manifestations included Addison disease (on hydrocortisone and fludrocortisone since 12 years old), chronic mucocutaneous candidiasis, hypoparathyroidism with intermittent hypoglycaemia, primary ovarian failure, and obesity, consistent with APS1.

**Fig. 3:**
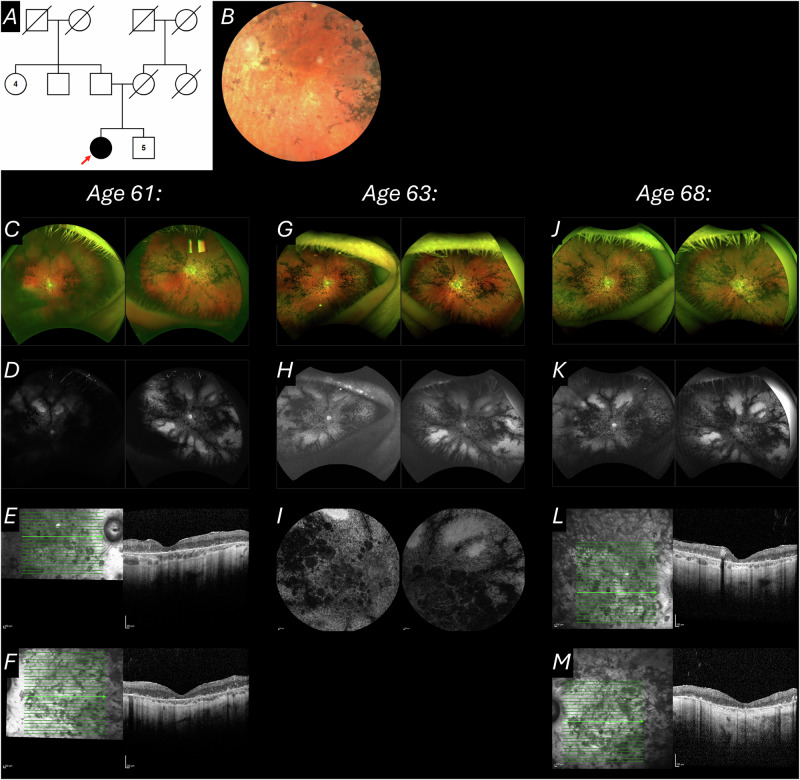
Pedigree & multimodal imaging from Case 4. **A** Family pedigree. **B** Left eye colour fundus image aged 52. Colour fundus (**C**) autofluorescence (**D**) and OCT (**E**, **F**) images from right and left eyes aged 61. Colour fundus (**G**) and autofluorescence (**H**, **I**) images from right and left eyes aged 63. Colour fundus (**J**) autofluorescence (**K**) and OCT (**L**, **M**) images from right and left eyes aged 68.

At 47 years old, her best-corrected visual acuity (BCVA) was 6/12 (20/40) in both eyes. Fundoscopy revealed paravascular pigment clumping and chorioretinal atrophy radiating from the optic disc. Her pedigree and fundus imaging (from 52 to 68 years of age) are shown in Fig. 3.

By 68 years old, she retained only light perception in both eyes (bilaterally pseudophakic). She was on systemic corticosteroids for adrenal insufficiency, and no additional immunosuppressive treatment was initiated.

The patient previously underwent targeted *AIRE* gene testing, which identified a homozygous pathogenic *AIRE* frameshift deletion: c.967_979del; p.(Leu323Serfs*5), a known disease-causing variant [13]. She also underwent whole genome sequencing, and no additional pathogenic genotypes were identified in known IRD genes.

#### Case 5

This case describes a 33-year-old woman with loss-of-function *AIRE* variants (Table 1). Her systemic phenotype conforms to the classical triad of APS1 and includes autoimmune ovarian failure and ectodermal features.

**Fig. 4:**
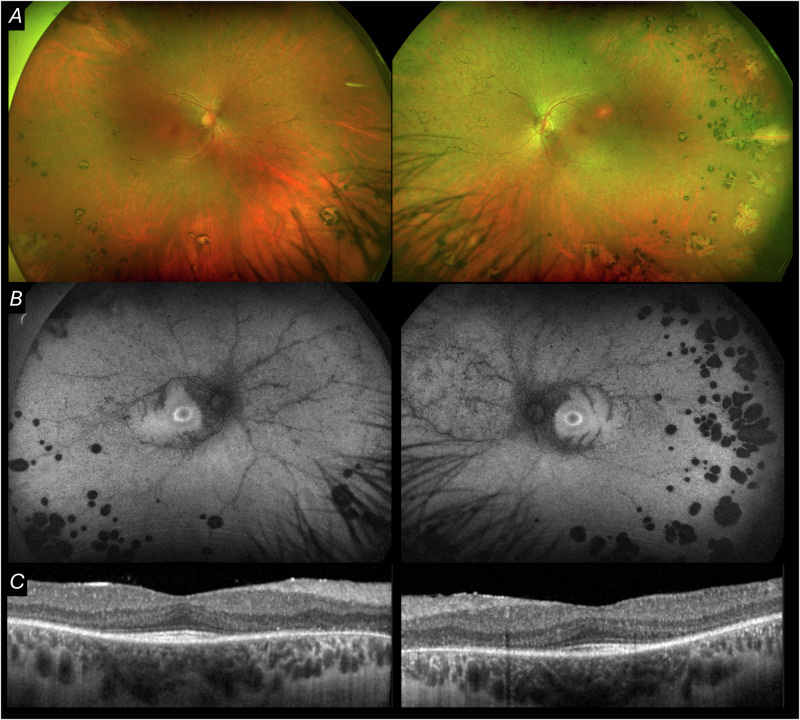
Multimodal imaging from Case 5. **A** Ultra-widefield pseudocolour imaging from both eyes. **B** Fundus autofluorescence showing characteristic hyperautofluorescent ring (“Robson ring”). **C** OCT demonstrating outer retinal thinning with preserved fovea from both eyes.

At 19 years old, she developed concentric visual field constriction. Fundoscopic examination revealed dense mid-peripheral bone-spicule pigmentation with relative preservation of the macula. OCT demonstrated parafoveal loss of the ellipsoid and external limiting membranes, with an intact foveal centre (Fig. 4C). FAF showed a hyperautofluorescent ring - resembling the “Robson ring” - demarcating the outer boundary of intact photoreceptors (Fig. 4A, B), seen in rod-cone dystrophies [14]. Progression of retinal degeneration was observed over time, with no evidence of anterior segment inflammation or keratopathy.

This case has previously been reported by Breunig et al. in 2013. However, from 2019 to 2023, the patient received B-cell-depleting therapy with rituximab, administered biannually [7]. Visual acuity remained stable under this regimen. However, rituximab was discontinued due to episodes of neutropenia. Subsequent treatment with adalimumab was curtailed following the emergence of psoriatic dermatoses.

The patient is currently maintained on cyclosporine (100 mg daily), mycophenolate mofetil (750 mg twice daily), and low-dose prednisone. Best-corrected visual acuities are 20/25 (OD) and 20/30 (OS). Despite treatment, OCT reveals ongoing parafoveal thinning.

### Previously reported cases

A literature search revealed 9 previous publications (reporting a total of 34 cases) describing retinopathy in association with APS1. Demographics, phenotypic features, genotypes and clinical course are summarised in Supplementary Table 1.

## Discussion

This case series contributes to the growing body of literature characterising the ocular manifestations of AIRE deficiency and underscores the marked phenotypic heterogeneity of *AIRE*-associated retinopathy. Our five cases with a median follow-up of 14 years, depict a wide spectrum - from preserved retinal architecture to a phenotype resembling severe rod-cone dystrophy - highlighting the unpredictable nature of this autoimmune retinopathy and possible benefit of therapeutic intervention in selected phenotypes.

Recent imaging and molecular studies have challenged the notion that *AIRE*-related retinopathy is extremely rare in this condition or phenotypically homogeneous. Bourgault et al. documented retinitis pigmentosa-like changes among five patients with APS1, with most demonstrating elevated serum antiretinal antibodies [5]. Complications such as retinal detachment following Vogt–Koyanagi–Harada-like uveitis, and fulminant choroiditis presenting in early childhood have also been reported [15, 16]. Retinal involvement is often characterised by peripheral RPE changes, sometimes accompanied by macular atrophy. Subsequent investigations have broadened this spectrum to include cone-predominant degeneration and multifocal choroiditis [7, 14].

Case 3 exemplifies a multifocal choroiditis-like phenotype with gradual progression over five decades, similar to the intermediate phenotype reported by Breunig et al. [7]. Unlike the treatment-refractory patients reported by Bourgault et al. [5], this individual retained central vision into late adulthood, which might be attributable to sustained therapeutic response to periocular corticosteroids This might suggest an optimal window for intervention - particularly when inflammation predominates - potentially altering disease trajectory. In contrast, Case 4 represents a phenotype with widespread central and peripheral retinal involvement, showing marked paravascular degeneration in the far periphery. No immunomodulatory treatment was attempted, and vision deteriorated to light perception. This case aligns with the progressive degeneration described by Sakaguchi et al. and supports histological findings of perivascular pigment accumulation and photoreceptor loss in APS1 donor eyes [8, 17].

In this series, patients with biallelic null *AIRE* variants exhibited markedly diverse phenotypes. Notably, the only two individuals with identical genotypes (Cases 1 and 4) had contrasting retinal outcomes, ranging from normal anatomy (Case 1) to severe vision loss (Case 4). This phenotypic discordance supports the influence of genetic modifiers (e.g. HLA haplotype, co-inherited inherited retinal disease-associated variants) or environmental factors (e.g. vitamin A status, viral mimicry), as previously proposed by Bourgault and Badawi [5, 18]. Some phenotypic features resemble other (sometimes variably defined) entities in the literature. Case 2 had features resembling AZOOR (acute zonal occult outer retinopathy), given the zonal outer retinal/RPE atrophy centred on the optic disc. Case 3 showed features in the right eye resembling multifocal choroiditis, but the peripheral degeneration was more confluent in the left eye. The degeneration in Case 4 was more widespread, affecting both central and peripheral retina. In the far peripheral retina, hypoautofluorescence was most marked around the vessels, the latter showing some similarity to findings in pigmented paravenous chorioretinal atrophy (PPCRA). In Case 5 the peripheral degeneration, central sparing and presence of the hyperautofluorescent ring bilaterally are all features also seen in rod-cone dystrophies. Recently, the entity of “Acute Outer Retinopathy” (AOR) has been described in detail by Ramtohul et al., who reported findings in 38 eyes: early photoreceptor disruption occurs leading to atrophy, with most progression occurring within initial weeks followed by stabilisation. They proposed that AOR can encompass a spectrum of conditions that include cases of acute annular outer retinopathy (AAOR), some types of autoimmune retinopathy, post-viral retinopathies and some cases of AZOOR. There is clear overlap between some of the findings in the present series and the phenotypic spectrum of AOR, although several cases in the present series showed chronicity and progression beyond the initial weeks. Future studies investigating early multimodal findings in *AIRE*-associated retinopathy could help more precisely delineate similarities and differences with respect to AOR.

Insights from animal models reinforce a central autoimmune mechanism. AIRE-deficient mice develop retinal infiltration by CD4⁺ and CD8⁺ T cells, targeting interphotoreceptor retinoid-binding protein (IRBP) [19]. Human studies confirm the presence of circulating antiretinal antibodies in 67% of APS1 patients, with specificity for recoverin, α-enolase, GAPDH, and other retinal proteins, mirroring features of non-paraneoplastic autoimmune retinopathy (AIR) [5, 7, 17]. Bourgault et al. similarly demonstrated serological immunoreactivity in most tested APS1 patients [5]. It should also be noted that circulating antiretinal antibodies are not specific; they have been shown to be present in some healthy individuals and also in individuals with other (non-autoimmune) retinal pathologies, where they might represent an incidental finding (or alternatively might mediate additional damage) [20].

Case 2 provides histopathological findings of relevance. Postmortem analysis revealed sharply demarcated zones of outer retinal, RPE, and choriocapillaris loss without inflammatory infiltrates - consistent with anatomically limited yet histologically destructive autoimmune retinopathy. It shows outer-retinal dropout and pigment migration identical to advanced RP, supporting photoreceptor loss rather than primary vascular ischaemia. The histology did not show active inflammation, though there was evidence of a type 1 CNVM. This case might suggest that treatment with immunosuppressive agents could be of benefit. However, the lack of activity might also represent end-stage or spontaneously quiescent disease rather than necessarily a response to treatment (this patient only had replacement steroids).

Case 5 offers supportive evidence for a B-cell-mediated mechanism. The patient experienced four years of disease stabilisation on rituximab, with apparent halting of constriction of the autofluorescent ring and visual field loss. Relapse following discontinuation and intolerance to TNF-α blockade highlight the immunological complexity of APS1 and the potential need for sustained therapy. However, as this is a single case-experience, broader applicability is not certain.

Collectively, our findings support a two-step model of disease development: AIRE deficiency permits autoreactive T and B cells (step one), leading in some cases to a slow progressive photoreceptor loss (step two). The abrupt transition between intact and atrophic retina, as seen in Case 3, might suggest that early immunomodulation could interrupt this process before central vision is compromised. Conventional corticosteroids alone have shown limited efficacy, as in the cases described by Wood et al. and Bourgault et al. [5, 21, 22]. In contrast, targeted immunotherapies such as rituximab have shown promise, though risks of immunosuppression (e.g. neutropenia, paradoxical inflammation) must be carefully balanced, and maintenance therapy may be required.

Limitations of this study include its retrospective design, small cohort size, and variable follow-up intervals, as well as inconsistent availability of treatment schedules and investigations including visual fields and electrophysiology. Antiretinal antibody testing was only undertaken in case 3; in the remaining cases, such testing was not performed and hence not available due to the retrospective nature of the case note review, which consequently limited the scope for correlative analyses and for verifying disease mechanisms. As discussed above, presence of antiretinal antibodies can also be non-specific and might not definitively prove aetiology. Cases were obtained from retinal specialists at ophthalmic centres, and focused on retinopathy; thus, the overall prevalence of retinopathy in APS1 remains unknown.

Given the variability in age of onset and rate of progression, lifelong surveillance is important for APS1 patients. High-resolution OCT and fundus autofluorescence can detect subclinical retinal changes before significant functional loss, potentially defining a therapeutic window. We suggest a protocol of baseline imaging at APS1 diagnosis, annual follow-up with multimodal imaging, and consideration of screening for serum antiretinal antibodies. Future research should prioritise biomarker identification and consider prospective trials of targeted immunotherapies. The development of standardised screening protocols and outcome measures will be essential for advancing clinical care and research in this rare but vision-threatening manifestation of APS1 and possibly for other primary immune regulatory disorders.

## Summary

### What was known before:

- Prior reports suggest a variable phenotype, ranging from mild retinal changes to severe vision loss, often presumed untreatable.

### What this study adds:

- The variability of the retinal manifestations the fact that vision loss is NOT inexorable, which is what is in the literature. Treatment prevents vision loss if used properly and in a timely fashion.

## Supplementary information

Supplementary Figure 1

Supplementary Table 1

## Data Availability

Data supporting the findings of this study are available from the study authors on request.
