# Supplementary material for: Retinopathy caused by a primary immune regulatory disorder - the spectrum of AIRE-associated retinopathy: case series and literature review
Source: Eye (Lond). 2026 Apr 9;40(9):1378–86. doi: 10.1038/s41433-026-04365-9 (PMC13269743; doi:10.1038/s41433-026-04365-9)
Supplement: Supplementary file 1 — Supplementary Figure 1 [file 41433_2026_4365_MOESM1_ESM.pdf]

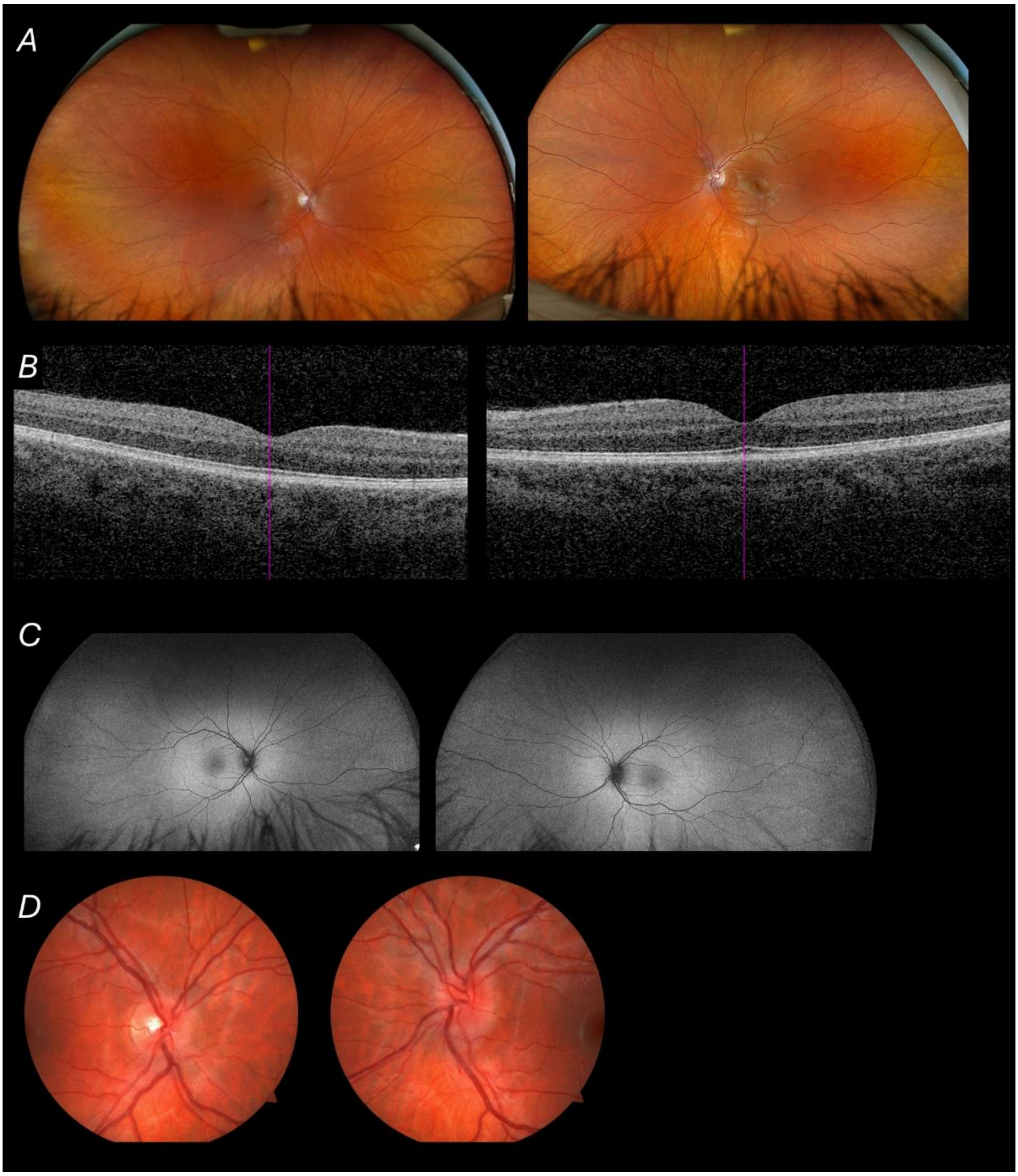

Supplementary Figure 1. Multimodal imaging from Case 1 of both eyes. A, Ultra-widefield pseudocolour fundus images at age 19. B, OCT images. C, Autofluorescence imaging. D, Colour fundus images at age 17.
