# Supplementary material for: Retinopathy caused by a primary immune regulatory disorder - the spectrum of AIRE-associated retinopathy: case series and literature review
Source: Eye (Lond). 2026 Apr 9;40(9):1378–86. doi: 10.1038/s41433-026-04365-9 (PMC13269743; doi:10.1038/s41433-026-04365-9)
Supplement: Supplementary file 2 — Supplementary Table 1 [file 41433_2026_4365_MOESM2_ESM.docx]

| **Publication** | **Study Design** | **Age (years)/ sex** | ***AIRE* variant(s)** | **Anti-retinal antibody result** | **Retinopathy cases / total** | **Key retinal phenotype** | **Diagnostic tests used** | **Other ocular findings** | **Major systemic APS1 features** | **Treatment & outcome** |
| --- | --- | --- | --- | --- | --- | --- | --- | --- | --- | --- |
| Wood 1991, USA [15] | Case report | 39M | Not sequenced | Not tested | 1 / 1 | Bilateral optic atrophy and bone spicule retinal pigmentary changes | Fundus photography, perimetry | None | Hypoparathyroidism, Addison's disease, candidiasis | Oral steroids: no improvement - 20/400 OD and 20/40 OS |
| Breunig 2013, USA [13] | Case report | 19F | c.769C>T; p.(Arg257*)/c.967_979del; p.(Leu323Serfs*51) | Enolase & GAPDH positive | 1 / 1 | Hyper-AF ring, flat mfERG | AF, OCT, full- & mf-ERG, GVF | Mild vitritis | Hypoparathyroidism, Addison disease | Prednisolone + CSA + MMF; fields improved |
| Bourgault 2015, CAN/USA [5] | Series (5) | 8-57 y, 3F/2M | Homozygous c.1616C>T; p.(Pro539Leu)  c.769C>T; p.( Arg257*)/c.967_979del; p.(Leu323Serfs*51)  homozygous c.967_979del; p.(Leu323Serfs*51)  c.769C>T; p.(Arg257*)/c.967_979del; p.(Leu323Serfs*51)  homozygous c.463G>A; p.(Gly155Ser) | 4 / 5 positive (WB/IHC) | 5 / 5 | Peripheral pigmentary change ± macular atrophy | AF, OCT, ERG | None | Variable triad | Various immunosuppressive agents; all cases progressed |
| Wang 2021, China [16] | Cohort (13) | 4-18 y, 8F/5M | 7 variants (4 novel):  c.38T>C; p.(Leu13Pro)  c.206A>C; p.(Gln69Pro)  c.269A>G; p.(Tyr90Cys)  c.489dup; p.(Lys164Glnfs*53)  c.623G>T; p.(Gly208Val)  c.739del; p.(Arg247Alafs*131)  c.922C>T; p.(Leu308Phe) | Not tested | 2 / 13 | One RP; one pigment + nystagmus | Fundus ± OCT | Keratitis (1) | Candidiasis, hypoparathyroidism | No ocular therapy |
| Sakaguchi 2021, Japan [17] | Case report | 2F | Homozygous c.415C>T; p.(Arg139*) | Recoverin positive | 1 / 1 | Severe rod-cone degeneration, hyper-AF ring | Wide-field AF, OCT, ERG | None | Autoimmune hepatitis | Pulsed methylprednisolone followed by prednisolone and azathioprine for hepatitis, with no effective retinal treatment |
| Culp 2022, USA [18] | Case Report | 25F | Homozygous c.769C>T; p.(Arg257*) | Not tested | 1 / 1 | Right disc swelling; outer retinal atrophy and pigmentare change; histology: outer-retinal and RPE atrophy (see Fig. 2) | Fundus photos, histopathology of donor globes | Corneal epithelial erosisons, cortical cataracts | Addison disease, hypoparathyroidism | Already on fludrocortisone and corticosteroids. Given acetazolamide and underwent right cataract surgery; presenting acuities 20/125 OD, 20/50 OS, with subsequent improvement |
| Bari 2023, India [19] | Sibling series (3) | 7-9 y, 2F/1M | Homozygous c.1A>G; p.Met1? | Not tested | 1 / 3 | Peripheral RPE loss, macular thinning | Wide-field AF, OCT | None | Alopecia, nail dystrophy | Lubricants; acuity 20/20-20/40 |
| Badawi 2023, Saudi [14] | Case Series (7) | 3-25 y, 3F/4M | Homozygous c.205_208dup; p.(Asp70Alafs*148) ± *PDE6C* c.481-1G>A; p.? | Not tested | 4 / 7 | Cone dystrophy, bull's-eye maculopathy, foveal thinning, ellipsoid zone loss | Full-field ERG, SD-OCT, FAF, fundus photography | Autoimmune keratitis, corneal opacities | Addison disease, hypoparathyroidism, hypothyroidism | Topical cyclosporine A 1%; vision varied (20/80-NLP) |
| Wei 2023, China [20] | Case report | 3M | Homozygous c.769C>T; p.(Arg257*) | Not tested | 1 / 1 | LCA-like with widespread retinal depigmentation, undetectable ERG | Fundus, AF, OCT, ERG | None | Dental enamel, nail dystrophy | Supportive; vision <20/400 |

Supplementary Table 1: Published cases of *AIRE*-associated retinopathy, including the specific variants, testing and treatments undertaken.

AF, autofluorescence; CSA, cyclosporine A; ERG, electroretinography; FAF, fundus autofluorescence; GVF, Goldmann visual fields; IHC, immunohistochemistry; IS, immunosuppression; LCA, Leber congenital amaurosis; mfERG, multifocal electroretinography; MMF, mycophenolate mofetil; NLP, no light perception; OCT, optical coherence tomography; OD, right eye; OS, left eye; Pred, prednisolone; RP, retinitis pigmentosa; RPE, retinal pigment epithelium; SD-OCT, spectral-domain optical coherence tomography; WB, Western blot
